# Silicate Fillings*Read before the Chicago Dental Society, March 19, 1918, and reprinted from the *Dental Review* for June, together with the discussion made by Dr. W. V-B. Ames.

**Published:** 1918-06

**Authors:** Edmund Noyes

**Affiliations:** Chicago, Ill.


### SILICATE FILLINGS*

BY DR. EDMUND NOYES, CHICAGO, ILL.

The time appears to have arrived when it can be affirmed that silicate fillings have acquired a positive and useful, and probably a permanent place in dental practice. Perhaps this has become so apparent to everybody that it is hardly worth while to mention but it has not been such a very long time that this could be said. Ten or fifteen years ago it would, perhaps, have been generally con-

* Read before the Chicago Dental Society, March 19, 1918, and reprinted from the *Dental Review* for June, together with the discussion made by Dr. W. V-B. Ames.

ceded that the use of porcelain had acquired a permanent place in general practice but we have seen a gradually diminishing use until it is almost limited to a few who have acquired special skill in making porcelain inlays, some of whom almost make it a specialty, and its use for jacket crowns, which are more frequently made than formerly. Whether there will be a similar rise and fall in the use of the silicate filling I will not venture to prophesy; suffice it to say that neither the porcelain inlay nor the silicate filling can fairly be called a *fad* for they both help to supply an important and urgent need in the practice of dentistry.

The chemical composition of the ingredients out of which a silicate filling is made is pretty well known to the chemists, but the makers of them profess not to understand perfectly the chemical character of the hardened material; the same is true of porcelain after baking. There can be no doubt that the chemical reactions taking place during the mixing of the powder and the liquid and subsequently in both the primary and secondary hardening have a determining relation to the laws that should govern the mixing, the insertion into a cavity and the care of the operation subsequently required, and the makers of these materials have given detailed instructions which they insist must be carefully followed if the best results are to be obtained. These instructions are based in part upon a knowledge of the chemical reactions taking place and the time required for their completion, and partly upon observation and experience in the use of the material. The silicates contain many of the same ingredients as porcelain which accounts for their similarity in appearance. Dr. Vogt says the initial hardening is analogous to the setting of an oxy-phosphate of zinc cement. The secondary hardening is radically different and "seems to be due to the same general causes as the setting of Portland cement." "While the silicate is in the plastic state it is *not* hydraulic. This is due to the fact that the formation of the insoluble hydrated phosphates from the soluble acid phosphates requires some time for its completion. Hence in order to avoid the solvent action of moisture at this stage some precautions should be observed.

1. The tooth should be isolated by the rubber dam.

2. The cavity should be dry. These simple methods will prevent any solution of the soluble salts before they have had the time necessary to convert them into insoluble ones.

3. Inasmuch as the silicate undergoes a secondary hardening accompanied by a marked change in physical properties, in order to obtain the best possible results the filling should be coated with a varnish or other waterproof coating which will prevent moisture from interfering with its proper crystalization.''

''One would not use a garden hose on a cement walk until at least twenty-four hours after it had been placed, so why should he permit water to come in contact with a silicate filling, the secondary hardening of which is analogous to that of Portland cement, until that filling had been allowed the same length of time for the final step in its hardening.'' The preceding quotation is from a paper by Dr. C. C. Vogt read at the meeting of the National Dental Association. Dr. Vogt conducted the experimental and research work at the Mellen Institute at Pittsburg which resulted in the production of the silicate filling material known as Certified Enamel. The hardening of a silicate filling does not appear to be a crystallization to any great extent and that is said to explain its translucency.

It is understood that this is to be a short paper, but it seems worth while to describe briefly the more important steps in the making of a silicate filling. The cavity should be of retentive form, for at least two of the silicates in common use, Ascher's New Enamel and De Trey's Synthetic Porcelain, are not sticky when properly mixed. Dr. Ames' Berylite and the new Certified Enamel are sticky and have some adhesiveness to the walls of the cavity but a fairly retentive form is desirable for these. The cavity margins should not be beveled; this material will not retain a feather edge as gold will. It is more like amalgam in this respect. Some authorities insist that all cavities should be lined with varnish and one writer assigns as a reason that too dry a cavity may absorb water from the filling, or if not dry enough may give up moisture to the filling, in either case to its detriment. Perhaps the point is too fine, and probably the question of cavity lining is not really different from what it is under amalgam or gold fillings and relates chiefly to protection of the pulp in deep cavities. If varnish is used the cavity margins should be trimmed afterward to make sure that no varnish remains on the enamel portions of the cavity walls. While the question of moisture either into or out of the cavity walls is probably too fine to be of practical importance, it is undoubtedly of great importance that the accurate balance and proportion of ingredients be preserved, especially as regards the water in the liquid and the possible absorption of moisture by the powder, and especially, for here the difficulty is greater, the absorption or the evaporation of moisture by the surface of the filling, either during the first setting or the secondary hardening during a number of hours after insertion. The bottles should be unstopped only long enough to get out what is wanted.

All authorities agree that the greatest care must be taken to have everything used in the handling of the silicates scrupulously clean. The mixing slab, the spatula, and the filling instruments must be cleaned with the greatest care. There are two reasons slight contaminations may interfere more or less with the chemical reactions in hardening and setting, and what is probably of more practical importance, very minute amounts of foreign matter may injure the shade of color.

The completeness of the chemical reactions in the filling require that the materials be very thoroughly spatulated (not merely mixed), for some time, and this can only be done sufficiently on a cold mixing slab, say from 55 to 65 degrees of temperature, if colder than 55 or 60 degrees it may prove painful in a sensitive cavity. By far the best way to manage this temperature proposition is to use a square bottle with a thermometer in it, then you know what temperature you have got and it will stay cool longer than a slab. The most satisfactory plan is to have a hygrometer and a dew-point table. (The hygrometers are usually sold with a humidity table but it is a dew-point table that we want. They are supplied by Dr. Ames.) When nearly ready the assistant notes the difference between the wet and dry bulbs, finds the dew-point by reference to the table, and takes the mixing bottle to the wash bowl and fills it with water at least two or three degrees above dew-point. During that part of the year when there is no artificial heat in our offices the dew-point will often be found above sixty or even sixty-five degrees and I have known it to be above seventy. When as high as that it is desirable, if practicable, to postpone the making of silicate fillings till a more favorable time. If neither the hygrometer nor the mixing bottle with thermometer inside are used, the slab may be cooled under the cold water faucet or in ice water and then after thoroughly drying, breathe on it and if the moist area contracts, showing evaporation from the slab it is above the dew-point and may safely be used, but if the moisture from the breath does not show any drying the slab is too cold and a filling mixed on it would probably be spoiled. At best this method is a guess as to the temperature of the slab. If you are going to make silicate fillings at all why not equip yourself to make them right?

For mixing the ingredients a spatula of bone or ivory or hard glass may be used but I believe one of agate is much preferable. It is easily cleaned perfectly and is hard enough not to be scratched. One man in discussing this subject advised a mixing slab of agate for the same reasons. The convenience of the bottle with thermometer is important enough to counterbalance the slight disadvantage from the scratching of the glass by attrition from the spatulation. By far the best instruments for filling are those having tantalum points, though instruments of agate, bone, or ivory have been much used. It is desirable, if not indispensable, as in making gold or amalgam fillings, that instruments be of such form and size that they may reach all portions of a cavity. It was long affirmed that steel instruments must not touch the material till after it is hardened but lately it has been asserted that steel instruments may be used if kept smooth and brightly polished. This is difficult for the material before it hardens tarnishes and rusts steel rapidly.

The material should remain on the mixing bottle till carried to the cavity. The natural warmth of the tooth will cause much more rapid hardening in the tooth than on the slab. It may be hastened still more by the application of heat but it is said to be desirable that hardening begin at the surface of the dentine and proceed outward and therefore if heat is applied, it should be to the tooth instead of the surface of the filling. For proximal cavities a simple matrix may be made with a strip of celluloid or glazed linen tape passed between the teeth and held on the lingual side with the finger. When the cavity is full, a little over full, the tape is drawn over to confine the filling, which may be manipulated under the tape for contour and contact point, and held firmly in place till hard, usually five minutes or more. The filling may then be roughly trimmed, leaving some surplus everywhere if possible (if the bite interferes there can, of course, be no surplus at that point). The whole surface of the filling should be made as perfectly water-proof as possible by a coating of varnish or melted paraffin to last at least a few hours. If there should be only slight defect in the water-proof coating the injury resulting may possibly be limited to the surface and removed in finishing. Probably slight changes in the material continue for weeks and months but are so slight as to be negligible and after about twenty-four hours, perhaps less time, it becomes hydraulic and is better for the moisture of the mouth. You have probably inferred this from the keeping of the synthetic shade guide in a bottle of water.

The final finishing should be done carefully and patiently, chiefly with rather fine carborundum stones or paper discs, cutting against the margins of the filling wherever practicable and not from the filling toward the tooth surface. Steel trimmers may be used to a slight extent if handled carefully. Final finishing may be done with fine strips, cuttlefish discs, and accessible portions with arkansas stones. Carborundum and arkansas stones should be kept wet and discs and strips lubricated with cocoa butter, paraffin or oil vaseline. Great care should be observed to have this finishing accurately done. A slight protrusion of the filling may escape observation unless the margin is tested with a fine point.

### DISCUSSION

Dr. W. V. B. Ames:

When we see the name of Dr. Edmund Noyes on a program there is the assurance that the subject will be treated conservatively. In this instance his opening sentence is, "The time appears to have arrived when it can be affirmed that silicate fillings have acquired a positive and useful and probably a permanent place in dental practice."

We have been accustomed to expect from as careful a man as Dr. Noyes, a paper made up largely of advised precautions. Only a few years ago the average instructions given out by makers and essayists were so hedged with cautions that, as a sample of the attitude of the profession, a member of our state society was led to ask me as to my Berylite, "Can the operator have an evil thought without influencing the behavior of the cement?"

With correction of tendency to discolor and mastery of setting to cure pulp destruction, these cements of necessity became popular. There has, however, been a statement to this effect, "Had it happened that zinc oxid itself was translucent, there would have been no need for the silicate."

Silicious cements could not have become really useful as well as popular had there not been the inherent possibility of greater integrity of mass when subjected to stress, abrasion and solvents, as compared to oxyphosphate of zinc.

Translucency alone could not have justified the popularity of the material. Translucency with greater integrity of mass does justify their extensive use.

Clinical experience has taught that oxyphosphate of zinc is less dependable upon proximal than upon occlusal surfaces. This fact easily indicates that the zinc cement has greater physical than chemical stamina. The chance of subjection to the products of fermentation and putrefaction in interdental spaces is great, while upon occlusal surfaces it is reduced to the minimum and therefore there is the clinical fact.

The agglutinating phosphate formed in a mix of oxyphosphate of zinc which binds the remaining oxid particles into a mass, is liquefied by those by-products, whereas the binder produced during the mix and setting of a proper silicious cement is proof against these destructive agents.

Given translucency, and integrity of mass, there is one other factor which goes a long way in justifying the use of these cements. This is the fact that the best texture, i. e. integrity of mass, may come from a consistency which will permit of almost any operation, from the making of a filling to the setting of a porcelain jacket crown. In other words, the consistency of mix which will enable the setting of such a crown is very little removed from that from which best results would be expected in the making of a filling.

For arriving at a proper proportion of powder and liquid in mixing these cements, I want to suggest the trying out by weight of one grain of powder to each drop of liquid as dropped from the sort of small pipette furnished with several of these cements. By such a trial it will be found that seven-eighths as much powder is required in a mix which is yet thoroughly plastic as is one which is slightly gummy. A slightly gummy mix is as stiff as need be for maximum integrity of mass, and the difference between this and the result of the mix sufficiently plastic for setting an inlay can not be easily distinguished.

I heartily commend Dr. Noyes' telling you to recognize that the initial hardening is analogous to the setting of oxyphosphate of zinc cement. Recognizing the silicious cements as oxyphosphates with which chemical behavior may be regulated to suit the requirements of the case, enables the operator to become master of the situation.

Just in proportion as silicious cements are looked upon as differing radically from other oxyphosphates in the chemistry of the setting process, will the manipulation present difficulties. By considering these cements as *silicious oxyphosphates* we get close to an understanding.

These cements *are* oxyphosphates of calcium and should be thought of as being so compounded that cement making phenomena come from action of phosphoric acid on calcium oxid, the action being retarded by the intimate blend *by fusion* with alumina and silica. The alumina and silica act as diluents and give integrity to the resulting mass.

The ingredients of the powders are very similar to a calcium feldspar. Feldspar furnishes the chief ingredients of porcelain and these cements might reasonably be considered a porcelain in which there is *chemical* agglutination, instead of by heat.

The term ''silicious cement'' appeals to some for descriptive purposes, rather than ''silicate.'' Normal silicates do not afford cement making properties. Silicious mixtures may.

Knowledge of some of the chemical reactions taking place should assure one of constant behavior. Variability of behavior has been the stumbling block, and this has been induced to a large extent by direct and indirect claims of manufacturers that the so-called silicates needed to be considered as differing radically from oxyphosphates of zinc.

The manufacturers of translucent and other cement have been much berated by essayists at times for refraining from disclosing the formulae and methods of manufacture as an aid to the dentist in the use of the material. On requests for such information, I have furnished the following:

''Ames' Berylite liquid is a modified phosphoric acid solution, and the powder is composed of basic silicates of calcium and aluminum, aluminates and rare earths combined at so high a heat that an indescribably complex compound results.'' Much camouflage, including patent specifications, has been offered which has not come as near the facts as my simple statements. Today it is pleasing to have Dr. Noyes present this subject, recognizing the material as oxyphosphate and without this sort of a grievance against the manufacturers.

Recognizing these materials as oxyphosphates, we have a tangible beginning toward understanding the primary setting process. More than twenty years ago I attempted to make plain the setting of oxyphosphates by assuming that they were masses in which oxid granules were held together by a basic phosphate. In the case of the silicious cements, or so-called silicates, the part corresponding to the zinc oxid of an oxyphosphate of zinc happens to be a powdered glass, which, as we have said, has been produced from fusing calcium oxid, silica, and alumina or closely allied compounds along with modifying ingredients at so high a heat that "an indescribably complex compound results."

No one risks his reputation in attempting to accurately describe the nature of such compounds, any more than writers on glass and glass manufacture attempt to definitely give the chemistry of any sort of glass, much less to venture a positive opinion on one requiring in its fusion the very high temperature needed for the fusion to a glass of the ingredients used for Berylite, for instance, in the proportion in which they are blended. (Samples of glass passed.)

For dental cement purposes this must be a basic glass instead of a true silicate and it is for this reason that I prefer the term "silicious cement" instead of "silicate cement." Basic silicates are possible cement ingredients, normal silicates are not. The basisity of such a glass enables the acid to attack it with the result of producing a cement.

The fineness of division of such a ground glass will affect the setting as well as the translucency. From fine grinding more surface of the basic glass is presented for action and quicker setting will result. In proportion to the fineness of grinding there is greater refraction of light and reduction of translucency. A happy medium is therefore possible in the grinding for both setting and translucency. An extra fine powder adapted to setting of inlays and porcelain jacket crowns will not give satisfactory translucency *in mass*, but is entirely satisfactory as a thin film.

After recognizing the mass resulting from treating such a glass by an acid phosphate solution, as an oxyphosphate, we can, with profit, theorize as to the nature of the material which forms during the mix and which agglutinates the undissolved particles of the basic glass to give a cement. Dr. Vogt before a section of the National Association, 1917, in accounting for the initial hardening of the cement, says that the soluble acid phosphates formed during the first part of the mix are converted into very insoluble normal phosphate, combine with water and crystallize out in so dense a mass of minute interlocked crystals that the individual crystals can not be detected even by a high power microscope. He later attributes the secondary hardening partly to "desiccation of colloids."

Without attempting a lucid explanation of colloidal structure and efficiency, I have, during more than twenty years, spoken of the binding together of the basic undissolved particles in cement as "agglutination." Glue is colloidal. A colloid is a glue or jelly-like substance. Chemically it is specifically uncrystalline. In subjecting the powdered basic glass to an acid phosphate solution, the phosphate formed from the action of acid upon base, passes, I believe, from the acid to the normal and on to a more basic and solid form, from being supersaturated by the base with which it is in contact.

Particles are thus bound together and the colloidal or glutinous phosphate which is *also* in contact with a surface of dentin or enamel, literally knits to the inequalities, i. e. the orifices of the tubili and any irregularities of the enamel surface and probably becomes basic to the extent of becoming a solid, *partly* by the acid becoming satisfied at the expense of tooth substance. Adhesion may be expected from this knitting to inequalities of dentin and enamel by such a glutinous substance. I derive more satisfaction from considering that this colloidal phosphate becomes a solid by supersaturation, rather than by desiccation, especially since desiccation is the thing to be avoided in the proper setting of these cements.

This is to me a more plausible explanation than that *normal* phosphate crystallizes out as crystals so minute as to be indistinguishable under the most powerful microscope, but as far as enabling an appreciation of and control of setting, it is tweedledum and tweedledee, whether the agglutinating substance is normal or basic phosphate.

Thinking of the phosphate as becoming basic at the expense of the basic glass helps me to a satisfactory explanation of the primary setting of cement and does not hamper my appreciation of the secondary setting.

Dr. Vogt in the paper before mentioned, laid special stress on extensive spatulation and cited that a hardened pellet can be crushed to a powder which will react with more of the cement liquid. This proves mostly that the binder is such that original particles are fractured in the grinding of the pellet, if it is conceded that any mass of cement consists of particles of powder held together by the phosphate formed during the mix. It is a case of the glue being stronger than the object glued. In the discussion of Dr. Vogt's paper I felt called on to caution against unlimited spatulation with *all* cements, as with some varieties enough *normal* phosphate may be produced to kill setting. Dr. Noyes advises extensive spatulation. I need to say that an amount of spatulation which would be needed with Certified Enamel would be ill-advised with Berylite, this because of a much heavier liquid being furnished with Berylite, i. e., it contains more phosphatic modifier and less water. Such cements should be spatulated to produce a homeogenous mass, but overspatulation is easily possible.

I have never considered arsenic to be the devitalizing agent in silicious cements, but have ventured the opinion that free phosphoric acid should be held responsible. It is largely with the object in view of getting rid of free phosphoric acid and causing nearly complete setting in a comparatively short time that I have so strongly advocated the application of heat.

Dr. Weston A. Price in 1914 ventured the opinion as a result of tests, that any temperature up to 175 degrees F. was permissible, shortening the time of setting, making a harder and stronger mass under crushing tests after twenty-four hours and in every particular seeming to make as good or a better filling. I am inclined to say that 125

degrees F. is a sufficiently high temperature for the purpose and is as high as can be conveniently imparted to a vital human tooth. There is no question in my mind but that with a cement in which the liquid has not too large a water content, acceleration of setting by heat is advantageous.

As to the proper time of finishing, there is no doubt of the advantage of waiting the twenty-four hours or more, as the texture is better by that time for taking a finish. As to the need of being kept dry, if caused to set promptly, I do not believe there is any real need of the mass being kept even partially protected. Complete dryness is not accomplished and if it could be, it would be objectionable. If the filling is left slightly overfull there is then material for the finish.

If it happens that a cement is peculiar in that it presents a better appearance some hours after its insertion than when primary setting only has taken place, a covering of paraffin or varnish is useful in providing for a better first impression to the patient than if inspected soon after insertion.

